# The Biological Effects of Polystyrene Nanoplastics on Human Peripheral Blood Lymphocytes

**DOI:** 10.3390/nano12101632

**Published:** 2022-05-11

**Authors:** Devojit Kumar Sarma, Ruchi Dubey, Ravindra M. Samarth, Swasti Shubham, Pritom Chowdhury, Manoj Kumawat, Vinod Verma, Rajnarayan R. Tiwari, Manoj Kumar

**Affiliations:** 1 ICMR—National Institute for Research in Environmental Health, Bhopal Bypass Road, Bhouri, Bhopal 462030, India; swasti.shubham@gmail.com (S.S.); manojkbiochem@gmail.com (M.K.); rajtiwari2810@yahoo.co.in (R.R.T.); 2 Department of Biotechnology, Barkatullah University, Bhopal 462026, India; ruchidubey90482@gmail.com; 3 Department of Research, ICMR—Bhopal Memorial Hospital & Research Centre, Bhopal 462038, India; rmsamarth@gmail.com; 4 Department of Biotechnology, Tocklai Tea Research Institute, Jorhat 785008, India; pritomc@gmail.com; 5 Stem Cell Research Centre, Department of Hematology, Sanjay Gandhi Post-Graduate Institute of Medical Sciences, Lucknow 226014, India; vverma29@gmail.com

**Keywords:** cytotoxicity, chromosomal aberration, DNA damage, environmental pollutant, genotoxicity, health, microplastics, micronucleus, nanoplastics

## Abstract

Environmental exposure to microplastics (MPs) and nanoplastics (NPs) is an increasing concern from human health perspectives. Little information on the genotoxic and cytotoxic potential of NP particles in human cells is available. We aimed to assess the cytotoxic and genotoxic potential of polystyrene nanoplastics (PSNPs) at different concentrations (2000μg/mL, 1000μg/mL, and 500μg/mL) by using chromosomal aberration (CA) and cytokinesis-block micronucleus assays (CBMN) on human peripheral lymphocytes. Dose-dependent hemolytic activity and cell viability were observed against the PSNPs exposure. Increased chromosomal aberrations, such as chromosomal breaks and dicentric chromosomes, and an increase in nucleoplasmic bridge (NBP) formation and nuclear budding (NBUD) were observed. The frequency of mitotic index (MI) decreased significantly in the PSNP-exposed groups from lower to higher concentrations. A significant increase in micronuclei (MN) formation and cytostasis% and a dose-dependent reduction in nuclear division index (NDI) in PSNP-exposed groups indicated oxidative stress-mediated cytotoxicity, DNA damage, and genomic instabilities due to PSNP exposure in human lymphocyte cells. This study highlights the importance of understanding the toxic mechanisms and associated chronic and acute health effects on humans due to exposure to this pervasive environmental pollutant.

## 1. Introduction

The increasing reliance of human life on plastic and its diverse products is evident from the rising trend of global plastic production since 1950 [1,2]. Approximately 360 million tonnes of plastic was produced globally in 2018 with Asian countries being the most prominent producers (>51%). Of the many different plastic types, polyethylene terephthalate (PET), polyethylene (PE), polypropylene (PP), polystyrene (PS), and polyvinyl chloride (PVC) are most widely used in consumer good products [3]. However, a large proportion of these plastics end up in the natural environment and landfills [1], posing a significant threat to human health and other living organisms [4,5].

Recently, overwhelmingly increasing evidence has showed the continuous exposure of humans and animals to microplastics (MPs, size ranging between 1 μM and 5 mm) and nanoplastics (NPs, size <1 μM) [6]. The source and origin of both MPs and NPs are ubiquitous in the environment, as most MPs are the product of continuous degradation and fragmentation of large plastics, in addition to the intentional addition of micron-sized plastic or fiber particles in many consumer products, such as facial scrubs, paints, textiles, etc. On the other hand, NPs are derived from the further degradation of MPs [7]. Due to their ever-presence in different environments, making humans vulnerable to frequent exposure via several known and unknown pathways, it is imperative to assess their possible harmful effects on human health [7].

Plastics are organic polymers composed mainly of carbon and hydrogen. Their physico-chemical properties and associated toxic effects can largely be attributed to the types, sizes, shapes, surface properties, solubility, and reactivity, all of which impact the biological activities within cells. Polystyrene microplastics (PSMPs) are one of the most predominant types of MPs present in the atmosphere [8]. NPs can enter living organisms through inhalation, ingestion via food and water, and also through dermal absorption [9,10]. Most airborne NPs can reach the alveolar surface and pass into the bloodstream, thereby initiating inflammatory reactions, leading to pulmonary fibrosis or even carcinogenesis [11,12]. Even the ingested MPs and NPs can be uptaken by gut enterocytes, accumulate in villi and release into the bloodstream, and reach other organs and tissues [13]. Recently the cytotoxic and genotoxic potential of different sizes of PSNPs has been studied in different cell lines and model organisms [14,15,16,17,18]. Due to the distinction in biological processes during differentiation and maturation, different cell lines display a disparity in biological activity against similar exposure [19]. Although our understanding of the effects (such as viability, DNA damage, toxicity, etc.) of MPs and NPs exposure on different human cells is gradually increasing [16,17,20,21,22], there is very little information on the toxic potential of NPs in human lymphocytes [18]. As the particle sizes decrease to nano-size, its surface-to-volume ratio increases, which makes them biologically more reactive and increases intracellular permeability [12,17,20,23,24,25,26,27]. Therefore, it is important to understand the biological effect of nano-sized plastic particles on human cells. To address this gap, we aimed to assess the genotoxic and cytotoxic potential of PSNPs with a size of 50 nm in human peripheral blood lymphocyte cells.

## 2. Materials and Methods

### 2.1. Chemicals

Cell culture grade reagents and chemicals were procured from the following suppliers: RPMI 1640 culture medium (Sigma-Aldrich Co. LLC, St Louis, MI, USA, Cat no.: R2405), Fetal Bovine Serum (HyClone™,Fisher Scientific, Hampton, NH, USA, Cat no.:SV30160.03), 1× Phosphate buffer saline (Gibco, Thermo Fisher Scientific, Waltham, MA, USA, cat no.: 10010-023), Phytohemagglutinin (PHA, Gibco, Thermo Fisher Scientific, Waltham, MA, USA, Cat no.:10576 015), Cytochalasin B (Sigma-Aldrich Co. LLC, St Louis, MI, USA, Cat no: C6762), Colcemid™ Solution (Gibco, Thermo Fisher Scientific, Waltham, MA, USA, Cat no.: 15210040), KCl (Sigma-Aldrich Co. LLC, St Louis, MI, USA, Cat no.: P5405), and Histopaque (Sigma-Aldrich Co. LLC, St Louis, MI, USA, Cat no.: 10771), 0.4% Trypan blue (Sigma-Aldrich Co. LLC, St Louis, MI, USA, Cat no.: 93595).

### 2.2. Polystyrene Nanoplastic

Polystyrene nanoplastics (PSNPs, Cat. No: 08691-10) with a diameter of 50 nm were purchased from Polysciences INC, Warrington, PA, USA. These particles were packaged as 2.5% solids (*w*/*v*) in an aqueous solution with a particle concentration of ~3.64 × 10^14^ particles/mL. The PSNP solution was first sterilized by UV exposure for 1 h before making stock solutions. To assess the genetic damage caused by the PSNPs, relatively higher concentrations for a short time exposure in cytogenetic assays were selected, as per the guidelines of OECD [28] and others [17]. Accordingly, the stock solution of PSNP was further diluted into 2000 μg/mL, 1000 μg/mL, and 500 μg/mL working concentrations in cell culture medium, to be used as exposure in different biological assays. These concentrations correspond to 29,120 × 10^9^, 14,560 × 10^9^, and 7280 × 10^9^ PSNPs particles, respectively.

### 2.3. Isolation and In-Vitro Culture of Lymphocytes

Peripheral blood samples were collected by venipuncture under sterile conditions in heparinized tubes from two healthy 25–35 years old volunteer donors with no history of smoking, drinking, or chronic drug use after obtaining written, informed consent. Peripheral blood lymphocytes were isolated using sterile, endotoxin-free Histopaque (Sigma-Aldrich Co. LLC, St Louis, MI, USA), following the method described by Panda and Ravindran [29]. Isolated lymphocytes were counted using a glass hemocytometer (Sigma-Aldrich Co. LLC, St Louis, MI, USA) [30].

### 2.4. Cell Viability Assay

The viability of the isolated lymphocyte cells exposed against different concentrations of PSNPs was checked following the Trypan blue exclusion method [31]. Briefly, 100μLof different concentrations of PSNPs were exposed to lymphocyte cells in RPMI medium and incubated (Model: Galaxy 170S, Manufacturer: New Brunswick, Eppendorf AG, Hamburg, Germany) for 24 h at 37 °C, 5% CO_2_. After centrifugation and the washing of cell pellets with 1× phosphate buffer saline (PBS), the cells were finally resuspended in 1 mL of 1× PBS (Thermo Fisher Scientific, Waltham, MA, USA). The cell suspension was mixed with an equal volume of 0.4% Trypan blue (Sigma-Aldrich Co. LLC, St Louis, MI, USA) and incubated at room temperature for 2–3 min. 10μLof this suspension was loaded into the hemocytometer (Sigma-Aldrich Co. LLC, St Louis, MI, USA), and both viable and dead cells were counted under a light microscope (DM200, Leica Microsystems, Wetzlar, Germany). Viability% was calculated using the following formula:

Viability% = (Number of viable cells/number of viable and dead cells) × 100.

### 2.5. Hemolysis Assay

The *in-vitro* hemolytic activity of PSNPs was assayed according to the previously described method [13,32]. Briefly, isolated erythrocytes were washed twice and diluted in 50 mL of 1× PBS. About 200 μL of erythrocyte suspension was transferred to 1× PBS (700 μL), containing the test concentrations of PSNPs, and incubated overnight at 37 °C. After incubation, the samples were centrifuged at 10,000× *g* for 5 min and the supernatants were transferred to 96-well plate. RBC suspension with 1× PBS is taken as the negative control and RBC suspension with distilled water is taken as the positive control. The absorbance values at 570 nm were recorded using an ELISA plate reader (Model: Elx800, Manufacturer: BioTek Instruments, Inc., Winooski, VT, USA). Hemolysis% was calculated by the following formula:

Hemolysis% = ((Sample absorbance − Negative control absorbance)/(Positive control absorbance − Negative control absorbance)) × 100

### 2.6. Chromosomal Aberration (CA) Assay and InVitro Cytokinesis—Block Micronucleus (CBMN) Assay

The standard methods were adopted for the preparation of the CA analysis and CBMN assay with minor modifications [33,34]. The cultures were set up in triplicates and two separate sets;one set was used for chromosomal analysis, while another set was used for MN assay. For chromosomal analysis, peripheral blood (0.5 mL) was added to 4 mL RPMI 1640 medium, supplemented with 20% fetal bovine serum and phytohaemagglutinin (PHA), and maintained at 37 °C for 72 h. After 24 h of culture initiation, the cells were exposed with different concentrations of PSNPs, as mentioned above, for 48 h after initiating the culture. The cells were treated with colchicine (0.06 μg/mL) 2 h before harvesting. At the end of the incubation, the cells were centrifuged at 1200 rpm for 15 min. Then, the cells were treated with 0.075 M KCl (37 °C) as the hypotonic solution and methanol:glacial acetic acid (3:1) as the fixative (at room temperature 22 °C ± 1 °C); the fixative treatments were repeated three times. The cells were centrifuged at 1200 rpm for 15 min after each fixative treatment. The staining of the air-dried slides was performed following the standard methods using 5% Giemsa stain (Cat no: 48900, Sigma-Aldrich Co. LLC, St. Louis, MI, USA) for CA. The mitotic index (MI), frequency of dicentrics, rings, chromatid breaks, and fragments were recorded. A total of 500 metaphases per individual were evaluated for CA and frequency was expressed as a percentage (%).

Another set was used for MN assay, in which peripheral blood (0.5 mL) was added to 4 mL RPMI 1640 medium, supplemented with 20% fetal bovine serum and phytohaemagglutinin (PHA), and maintained at 37 °C for 72 h. After 24 h of culture initiation, the cells were exposed with different concentrations of PSNPs, as mentioned above, for 48 h after initiating the culture. To block cytokinesis, cytochalasin B was added after 44 h of incubation at a final concentration of 6 μg/mL. After an additional 24 h incubation at 37 °C, the cells were initially harvested by centrifugation at 1200 rpm for 15 min and further processed identically, as described for the preparation of CA. The cells were hypotonically treated with 7 mL of cold (4 °C) 0.075 M KCl to lyse red blood cells and centrifuged immediately at 1200 rpm for 8 min. Finally, the slides were stained with 10% Giemsa. At least 500 binucleated lymphocytes from each individual were scored for MN frequency; MN frequency was expressed as MN/1000 (‰). A total of 1000 cells were scored to calculate the nuclear division index (NDI) or cytokinesis-block proliferation index (CBPI) using the formula:

NDI = ([1 × MI] + [2 × MII] + [3 × MIII] + [4 × MIV])/N,

where MI–MIV represents the number of cells with one to four nuclei and N is the total number of the cells scored.

In addition to NDI, the frequency of nucleoplasmic bridges (NPBs) formation and nuclear buds (NBUDs) as genomic alteration was also scored [20]. NPBs can originate from misrepaired DNA breaks or end fusions of telomeres, and can provide a direct measure of genome damage and chromosomal rearrangement [33]. NBUDs resulted from the nucleus as extroflections of nucleoplasmic material or as micronuclei connected to the nucleus by a bridge, and can be considered a biomarker for gene amplification [17,33]. Similarly, cytostasis, which occurs after the inhibition of cell growth and multiplication and is an overall measure of cytotoxicity, was also calculated according to [35], using the following formula:

Cytostasis% = 100 − 100 (CBPI*_T_* − 1)/(CBPI*_C_* − 1) (1)

where CBPI*_T_* and CBPI*_C_* represent cytokinesis-block proliferation index of PSNP-exposed and control cultures, respectively.

All assays were carried out in triplicates. Cell culture medium (RPMI-1640) was used as the negative control as the PSNPs concentration range was prepared in the same medium. Cyclophosphamide (HiMedia Laboratories Pvt Ltd., Maharashtra, India), being a potent human carcinogen [36], was used as a positive control at a concentration of 5μg/mL [34].

### 2.7. Statistical Analysis

All statistical analyses were performed with GraphPad Prism software (V8.0). The findings were presented as mean ± SEM and analyzed using one-way ANOVA followed by Tukey’s test, which was performed with the data from each experiment to assess statistical differences between groups. *p*-values less than 0.05 were considered statistically significant.

## 3. Results

**Figure 1:**
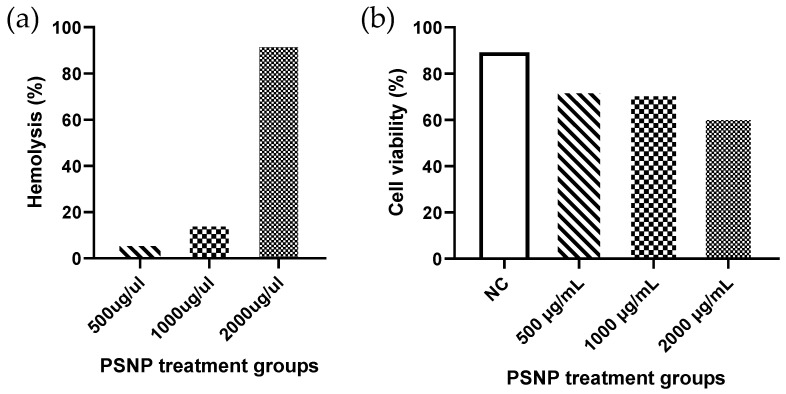
Percentage hemolysis (**a**) and cell viability (**b**) of lymphocyte cells treated with different concentrations of PSNP.

PSNP exposure affects the cell viability and hemolysis of lymphocyte cells in a dose-dependent way. Highest hemolysis (93%) was observed in the 2000μg/mL concentrations of PSNP exposure, in comparison to the other two doses (15.3% in 1000 μg/mL and 6.5% in 500 μg/mL) (Figure 1a). Similarly, a higher cell viability (73.1%) was observed in the lowest dose of PSNP. A substantial reduction in cell viability (39%) was observed in 2000μg/mL exposure (Figure 1b).

**Figure 2:**
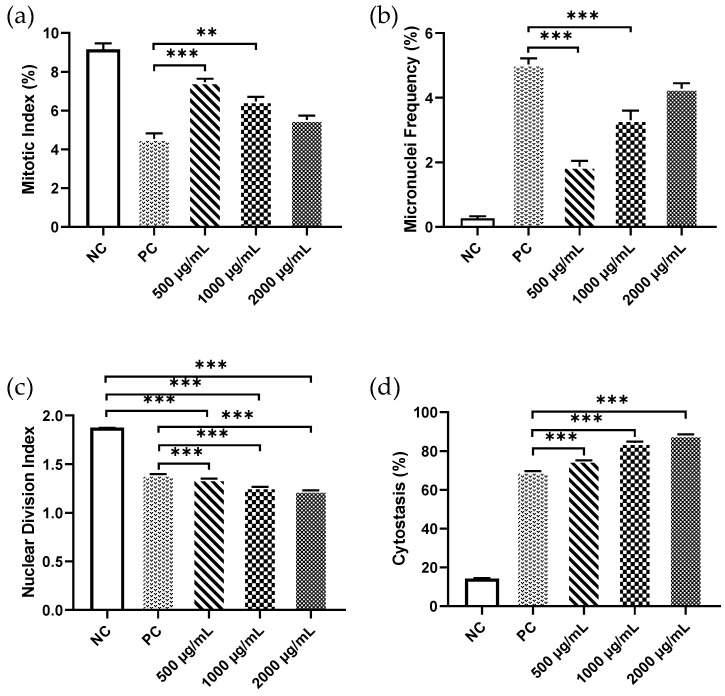
Mean values of (**a**) Mitotic index (MI), (**b**) micronuclei (MN) frequency, (**c**) nuclear division index (NDI) and (**d**) cytostasis % in lymphocytes exposed to different concentrations of PSNPs with a size of 50 nm in CBMN assays. Statistical significance was determined by one-way ANOVA followed by Tukey’s test. ** *p* ≤ 0.01, *** *p* ≤ 0.001. Error bars represent the standard error of mean.

In chromosomal aberration assay, the highest and lowest values of MI were observed in the negative and positive control groups, respectively. One-way ANOVA revealed a statistically significant difference in the mean MI values of PSNP-exposed groups (F = 51.64, *p* < 0.0001). A significant difference was observed for the mean MI values of 1000μg/mL (6.48 ± 0.22) and 500μg/mL (7.47 ± 0.18) PSNP-exposed groups, when compared to the positive control (4.54 ± 0.28), based on Tukey’s post-hoc comparisons. No significant difference was observed between the mean MI values of lymphocyte cells treated with 1000 μg/mL PSNP, when compared to the positive control (Figure 2a).

Increased chromosomal aberrations, such as chromosomal breaks, chromosome rings, and dicentric chromosomes, were observed in lymphocytes treated with different concentrations of PSNPs. The highest proportions of aberrant cells were observed in the group exposed with 2000μg/mL PSNP (Table 1).

Following treatment with PSNPs, a statistically significant increase in micronuclei (MN) frequencies were observed in a dose-dependent manner from lower to higher concentrations (R = 0.96, *p* = 0.042). The highest value for micronuclei frequency in bi-nucleated cells was recorded in the group exposed with 2000 μg/mL PSNP (4.28 ± 0.17, *p* < 0.0001), followed by 1000 μg/mL (3.31 ± 0.29, *p* < 0.0001) and 500μg/mL (1.87 ± 0.18, *p* < 0.01). Although the observed differences in MN frequencies against different treatment groups were statistically significant (F = 100.8, *p* < 0.0001), the difference in MN frequencies between the highest PSNP exposure group and the positive control group was not found to be significant (Figure 2b). We also observed an increase in frequencies of NPB and NBUD formation compared to the negative control. The formation of NPB and NBUD was also highest in the 2000 μg/mL concentration compared to the other 2 doses (Table 1).

A very highly significant value of NDI among and between different exposed groups was observed (*p* < 0.0001). The NDI values in the negative control were found to be 1.90 ± 0.03, whereas a significant decrease (*p* < 0.0001) in NDI (1.23 ± 0.02) was observed in the group treated with the highest concentration of PSNP (2000 μg/mL). The NDI values of the other two PSNP exposure groups were also found to be significantly lower than the control group. There was also a significant decrease in the NDI of the other three exposed groups when compared with the positive control (Figure 2c).

The cytostasis%, which is an indicator of cytotoxicity and occurs after cell growth and multiplication, was also found to have increased in a dose-dependent manner from lower to higher PSNP exposure (Figure 2d). The cytostasis% for all the PSNP exposure groups was found to be significantly higher than the control group, indicating prominent cellular toxicity of PSNPs on human lymphocyte cells.

## 4. Discussion

The ubiquitous nature of plastics in our environment and the formation of MPs and NPs due to the natural degradation of plastic or anthropogenic activities endanger human health to a great extent. The present study aimed to evaluate the genotoxic and cytotoxic effects of PSNP on human peripheral blood lymphocytes. We investigated cell viability, hemolysis, MI, NDI, MN, NPB formation, and NBUD to assess the biological effects of various concentrations of PSNP. We observed a dose-dependent increase in hemolysis, cytostasis, NDI, and MN frequencies from low to high dose exposure of PSNP, while the cell viability decreased as the exposure concentration increased. The frequencies of chromosomal breaks, dicentric chromosome, rings, NPB, and NBUD formation were also found to be most frequent in the highest PSNP exposure dose given.

We observed a steep rise in the rate of hemolysis at 2000 μg/mL PSNP exposure, which may be due to the physical damage to the RBC cell membrane caused by the aggregation of the nanoplastic particles at higher concentrations [17,20] and the endothelial adhesion of RBCs [37]. The concentration-dependent decrease in % cell viability observed may be due to the aggregation of PSNPs at higher concentrations, which tends to inhibit cell proliferation [17]. This may explain the decreased cell proliferation and increased cytostasis as the concentration of PSNP increases. A reduction in cell viability (40%) was observed at 1000 μg/cm^2^ concentration of PSMPs in BEAS-2B epithelial cells [12]. Similarly, reduced viability (24.82%) was also observed after exposure to PSNP with a size of 50 nm after 24 h of exposure in HepG2 cells [21].

The results of this investigation show a substantial, significant difference in NDI between the control and exposed groups. Increasing exposure concentrations also resulted in a significant rise in the frequency of MN and NBUD development. The frequencies of MN and NBUD formation were noted to be significant even in relatively lower doses (75 μg/mL) of PSNP with a size of 100 nm [17]. These findings imply that PSNPs can cause genotoxicity at a range of sizes and concentrations. The observed increase in the cytostasis induced by PSNP exposure on human lymphocyte cells indicates the inhibitory role of PSNPs on cellular proliferation. The observed cytotoxicity of PSNPs could be attributable to an increase in oxidative stress.

A significant increase in the accumulation of reactive oxygen species (ROS) has been found in both PSMPs (1000μg/cm^2^) and PSNPs (75μg/mL), facilitating reduced energy metabolism, tissue damage, and even genotoxicity [12,17]. It has been demonstrated that PSNP dose-dependently decreased superoxide dismutase (SOD) activity and glutathione (GSH) content, suggesting their role in the impairment of antioxidant capacities leading to cytotoxicity and cell death [21]. On the other hand, Xu et al. reported a rapid and efficient internalization by non-specific phagocytosis of PSNPs (with sizes of 25 nm and 75 nm) in alveolar epithelial cell, A549, which affects cell viability and induces cell cycle arrest. A significant up-regulation of pro-inflammatory cytokines and pro-apoptotic proteins indicated the involvement of TNF-α-associated apoptotic pathways in the toxicological effects induced by PSNPs [38]. Similarly, Wu et al. also reported the involvement of NF-κβ, MAPK signaling pathways in modulating cell inflammation and the proliferation in Caco-2 cells exposed against PSMPs, using a transcriptomic approach [39]. Hwang et al. [24] demonstrated that PS particles were not cytotoxic to human dermal fibroblasts and PBMCs in usual conditions, but might cause damage to the skin in extremely high concentrations. They also concluded that PSNPs with a size of <1μm affected RBCs and lead to hemolysis, which may be due to the adhesion of NPs by weak interactive forces, such as van der Waals forces, and due to its higher surface-to-volume ratio. These observations support the findings of the present study.

MPs and NPs are also regarded as carriers of potent endocrine-disrupting chemicals (EDCs), most of which are additives to plastics [40,41]. Phthalate esters, a prominent EDC, when added to NPs, exhibited significant toxicity at the higher concentration of NP in A549 cells [22]. This might be alarming, as an abundance of both NPs and EDCs are ubiquitous in the environment and specific target populations, such as industrial workers and residents of industrial areas, are at a high risk of exposure from both of these two emerging pollutants.

Human populations are generally thought to be exposed to low concentrations of NPs, in contrast to the people working in specific positions and places, such as in the plastic industry, at traffic points, etc., who may be exposed to high to very high concentrations of NPs, and this may lead to life-threatening occupational diseases. However, as the amount of plastic garbage generated increases, NP contamination in the environment may have an impact on the human community, as a whole. Recent studies have indicated the release of approximately 11.6 billion MPs and 3.1 billion NPs from a single plastic tea bag [42], at least 300 billion NPs/g from face scrubs [43], billions of NPs/mL (average size 160 nm) of water from polystyrene disposable cups [44], and an exposure of 14,600–4,550,000 MPs per capita per day from polypropylene infant feeding bottles [45], which are considered common sources of exposure to humans. The number of particles used for exposure in this study corresponds closely to the natural exposure of NPs in the human system; however, looking at the continuous exposure of NPs in the natural environment, these NPs may cause even more damage to human health than observed in the present study. Recently changes in cytotoxicity- and genotoxicity-related biomarkers were documented for chronic exposure of PSNPs (50 nm size) against Caco-2 cells, along with the accumulation of PSNPs in the cells, inducing changes at the ultrastructural and molecular levels [46]. Therefore, a more detailed investigation is required to identify the source and mode of exposure, quantify different types and sizes of NPs in humans, and assess their acute and chronic health impacts in different cell systems to understand their potential hazards as well as mechanisms.

## 5. Conclusions

According to the findings of this investigation, small-sized (50 nm) polystyrene nanoplastics have the potential to cause cytotoxic and genotoxic effects in peripheral human blood cells. The increasing frequencies of MN, NPB, and NBUD in the treated groups indicate the induction of chromosomal damage and genomic instability in lymphocyte cells due to PSNP exposures. Environmental exposure to smaller-sized NPs could effectively interact with the cellular system and block mitotic activities, leading to cytotoxicity and genotoxicity in human lymphocytes and thereby impacting human health. This study emphasizes the need to carry further research to better understand the toxicodynamics and mechanisms of NP induced cytotoxicity and genotoxicity by employing a range of cell models of various types, sizes, and functionally modified NPs, at environmentally relevant dosages.

## Figures and Tables

**Table 1:** Frequency of NPB, NBUD, and different chromosomal aberrations, such as chromosomal breaks, dicentric chromosomes, fragments, and rings in lymphocytes, treated with different concentrations of PSNPs after 48 h of exposure.

Concentrations (μg/mL)	NPB (%)	NBUD (%)	Chromosomal Breaks (%)	Dicentric Chromosomes (%)	Fragments (%)	Rings (%)
NC	-	-	2.12 ± 1.02	0.00 ± 0.00	2.16 ± 0.12	0.00 ± 0.00
500	2.4 ± 0.19	2.6 ± 0.18	3.88 ± 1.20	1.54 ± 0.46	3.46 ± 0.84	0.62 ± 0.46
1000	2.0 ± 0.27	3.4 ± 0.19	5.24 ± 1.34	2.64 ± 0.62	4.82 ± 1.96	1.28 ± 0.86
2000	3.6 ± 0.25	5.6 ± 0.29	6.48 ± 1.22	2.88 ± 0.68	6.46 ± 1.86	1.44 ± 0.88
PC	5.0 ± 0.22	7.2 ± 0.43	7.10 ± 1.42	3.42 ± 0.80	9.24 ± 2.12	1.62 ± 0.86

## Data Availability

Not applicable.
